# The Impact of Stroboscopic Visual Conditions on the Performance of Elite Curling Athletes

**DOI:** 10.3390/life14091184

**Published:** 2024-09-19

**Authors:** Tianhe Li, Chiyue Zhang, Xiaoyao Wang, Xinai Zhang, Zhiqiang Wu, Yapu Liang

**Affiliations:** 1 School of Strength and Conditioning Training, Beijing Sport University, Beijing 100084, China; 2023240685@bsu.edu.cn (T.L.); 2022240687@bsu.edu.cn (X.W.); 2023240662@bsu.edu.cn (X.Z.); 2 China Ice Sport College, Beijing Sport University, Beijing 100084, China; zhangchiyue2022@bsu.edu.cn

**Keywords:** stroboscopic visual conditions, elite curling athletes, curling performance

## Abstract

Background: In elite curling, precise time perception, speed control, and accuracy are critical components of performance. Stroboscopic training enhances visual processing speed, reaction time, motor skill control, and cognitive abilities by challenging the brain to make quick decisions with limited visual information. Purpose: This study aimed to investigate the impact of stroboscopic visual conditions on the key performance aspects of elite athletes in curling to determine whether these effects can be leveraged in long-term training to enhance elite curling performance. Methods: This study involved the participation of 32 national-level male curling athletes (*n* = 32, age: 19.9 ± 2.2 years, height: 178.0 ± 6.2 cm, body mass: 71.9 ± 10.6 kg, and training age: 2.7 ± 0.9 years). A cross-over controlled experiment was conducted, with participants randomly assigned to either a stroboscopic-first group (*n* = 16) or a control-first group (*n* = 16). Each participant completed tests under both stroboscopic and normal visual conditions, including assessments of time perception error, speed control error, and curling accuracy. Paired sample t-tests were employed to analyse performance differences across conditions, and two-factor ANOVA was used to analyse sequence effects. Bonferroni post-hoc tests were used to compare differences if the main effect was significant. Cohen’s *d* was used for two-group comparisons, whereas η_p_^2^ and Cohen’s *f* were used for comparisons involving three or more groups. Results: under stroboscopic conditions, participants experienced increased errors in time perception (*p* < 0.001, Cohen’s *d* = 1.143), delivery speed control (*p* = 0.016, Cohen’s *d* = 0.448), and reduced accuracy (*p* = 0.029, Cohen’s *d* = 0.404). The sequence main effect on speed control error was significant (*p* = 0.025, η_p_^2^ = 0.081, Cohen’s *f* = 0.297). Conclusions: Stroboscopic visual conditions negatively impacted cognition (especially time perception) and delivery performance focused on speed control and accuracy in elite curling, highlighting the potential and feasibility of using stroboscopic training to enhance elite curling performance.

## 1. Introduction

The sport of curling originated in Scotland during the 16th century. It has subsequently grown in popularity and is now a prominent feature of the Winter Olympics [1,2]. The game is played on a rectangular sheet of ice, with the primary objective being to slide granite stones towards a circular target area segmented into four concentric circles, known as the “house” [3,4].

The accuracy of stone delivery can be used as a performance indicator in curling. The precision of stone delivery is contingent upon the perception of time and regulation of the stone’s velocity. In the sport of curling, there is a strong correlation between time perception and performance [5]. Time perception encompasses the estimation of the timing of actions, as well as anticipation and preparation for the occurrence of events [6]. Furthermore, cognitive abilities, including time perception, can impact performance in time-constrained sports [7], particularly those requiring precise timing and accuracy, such as badminton, ice hockey, and others [7,8]. Additionally, movement can also influence or further enhance an athlete’s perception of time [9].

Furthermore, curling athletes must regulate the velocity of the stone in order to guarantee its adherence to the intended trajectory, thereby facilitating the attainment of tactical objectives or the scoring of points. The control of speed is contingent upon the acquisition and processing of information, which in turn gives rise to the execution of highly automated technical movements. Sensory perception (vision, proprioception, balance sense, and hearing) serves as the foundation for the reception of information pertaining to human motion, upon which technical movements are developed [10].

In recent years, the impact of visual training on athletic performance, especially regarding accuracy and cognition, has been the subject of considerable discussion and attention. Various studies have demonstrated that visual attention and occlusion training can significantly enhance perceptual abilities, anticipatory capabilities, and motor skills across a range of sports, including badminton, football, basketball, and motor racing [11,12,13].

Furthermore, stroboscopic visual training, which employs specific intermittent visual disruption frequencies, achieves visual occlusion in both temporal and spatial dimensions [14,15]. Firstly, stroboscopic training has been demonstrated to enhance visual–motor control and dynamic vision, with the potential to even improve jumping performance [16,17]. Additionally, visual occlusion training has been shown to reinforce athletes’ non-visual senses by disrupting visual feedback, thereby enhancing proprioception and vestibular function [18,19,20]. In the context of curling, it is of paramount importance to facilitate continuous enhancement in temporal perception and judgement, as well as muscle control and stability, in order to achieve speed control and enhance accuracy. This is closely linked with visual feedback, proprioception, and vestibular functions [21,22,23]. Nevertheless, no studies have substantiated the influence of stroboscopic visual conditions on curling performance, nor have they addressed the potential for visual training, particularly in relation to whether the cognitive and performance aspects of elite curling athletes are affected by stroboscopic visual conditions. Although research has validated the positive effects of stroboscopic training on cognition and performance in other sports, some controversy still exists [24]. Consequently, further investigation into the specific impact of stroboscopic visual conditions on high-level curling athletes is required.

This study aimed to investigate the impact of stroboscopic visual conditions on time perception and judgement, stone delivery speed control, and accuracy in elite curling athletes. This was achieved through a cross-over controlled experiment. In light of these findings, this study will inform the training of elite athletes, directing further research into the effects of stroboscopic training interventions and elite performance. This study investigated the specific impacts of stroboscopic visual conditions on the potential and feasibility of elite curling stroboscopic training, thereby deepening the theoretical understanding of visual training. This research hypothesised that stroboscopic visuals may disrupt the attention, anticipatory timing, and technical movements of elite curling athletes, thereby impairing their ability to perceive and judge time. This, in turn, may negatively impact delivery speed control and accuracy when compared to normal visual conditions. This hypothesis was based on previous studies in this field.

## 2. Materials and Methods

### 2.1. Subjects

This study involved the participation of 32 national-level male curling athletes (mean ± SD: male, *n* = 32, age = 19.9 ± 1.2 years, height = 178.0 ± 6.2 cm, body mass = 71.9 ± 10.6 kg, curling training age = 2.7 ± 0.9 years), as shown in Table 1. Athletes participating in this study were aged between 18 and 22 and engaged in curling-specific training at least seven times per week over five days. On training days, athletes engaged in one or two on-ice training sessions. In order to be eligible for inclusion in this study, participants were required to meet a number of specific criteria. The inclusion criteria required that participants regularly engaged in on-ice technical training, had a minimum of two years’ experience in curling, were right-handed, and possessed normal or corrected-to-normal vision. The exclusion criteria included whether participants experienced any sports injuries during the study, exhibited signs of infection or fatigue on the assessment day, had taken medications or drugs (including antidepressants and painkillers) on the assessment day, or consumed caffeine on the assessment day that could affect neuromuscular function during the tests. In addition, all participants were required to complete a medical history questionnaire in order to confirm their good health and the absence of any musculoskeletal, neurological, or other conditions that could affect their ability to perform curling techniques. Participant recruitment began on 1 January 2024 and ended on 7 January 2024. All subjects involved in this study signed an informed consent form. There were no statistically significant differences in these personal characteristics among the groups.

### 2.2. Ethical Approval

This study was conducted according to the guidelines of the Declaration of Helsinki and approved by the Sports Science Experiment Ethics Committee of Beijing Sport University (2024105H). Before the start of this study, all participants provided their written informed consent to participate after the benefits and potential risks were explained. The study was conducted according to the guidelines of the Declaration of Helsinki and approved by the Sports Science Experiment Ethics Committee of Beijing Sport University (2024105H). 

### 2.3. Experimental Protocol

**Figure 1:**
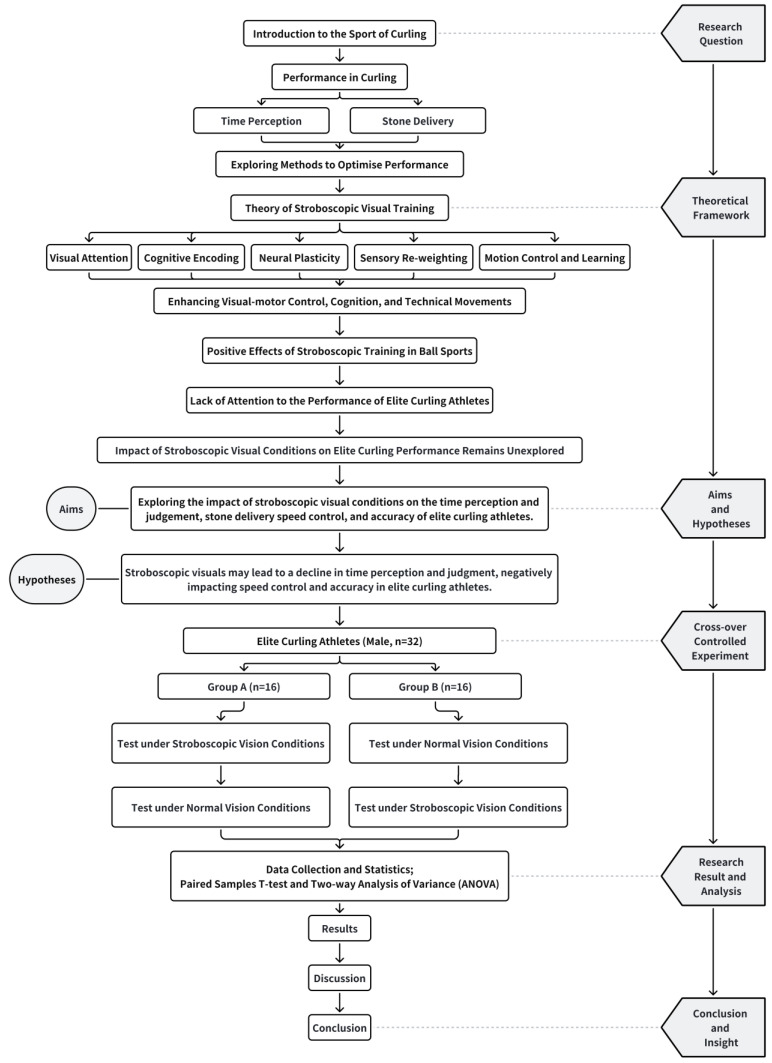
Research Framework. The figure illustrates the theoretical framework and design approach of this study.

**Figure 2:**
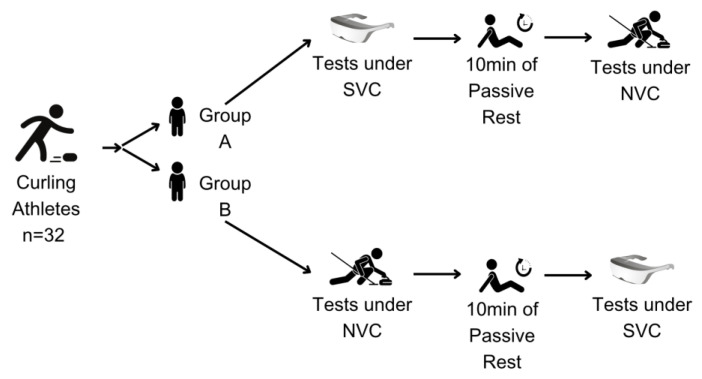
Experimental flowchart. SVC: stroboscopic visual conditions; NVC: normal visual conditions.

The principal aim of the initial phase of this investigation was to assess the impact of stroboscopic visuals on the performance of curling athletes, with a particular focus on their ability to control stone delivery speed and precision. A cross-over controlled trial methodology was employed in this study, which recruited a cohort of 32 participants. Subsequently, the participants were randomly allocated to either the stroboscopic priority group (Group A, comprising 16 individuals) or the control priority group (Group B, consisting of 16 individuals). Each participant was subjected to two distinct assessments conducted under disparate visual conditions. In particular, the individuals in Group A were initially evaluated under stroboscopic visual conditions (SVC), after which they underwent a passive interval of 15 min. They then underwent a second assessment under normal visual conditions (NVC). The frequency was set at 0.3 s of transparency, followed by 0.1 s of opacity. In contrast, the sequence was inverted for Group B. The assessments comprised evaluations of time perception in curling and on-ice curling performance, which included both the regulation of delivery speed and the precision of stone placement. The research framework is depicted in Figure 1 and the experimental procedure is delineated in Figure 2.

### 2.4. Time Perception and Judgment Test

Prior to the commencement of the time perception and judgment test, the experiment leader provided an explanation of the test content and tasks, followed by a practice session. Once the participants demonstrated proficiency in the experimental task, the practice session was brought to a close and the official test began. The participants were required to view video materials showing a curling stone moving in a defined pattern towards the hog line at the end of the playing area and then entering and covering a specified zone.

Subsequently, participants were required to ascertain the precise moment at which the front of the stone made contact with the T-line (highlighted in red in the video) and then press the button. It was required that each participant complete a series of judgement tasks on 10 randomly selected video materials. The discrepancy between each judgement time and the actual situation was recorded as a judgment error, with a precision of 0.01 s. A smaller average judgement error is indicative of enhanced temporal perception and judgement capabilities within the specified context.

This study employed the 3D modelling software 3D MAX 2020 (Autodesk, San Francisco, CA, USA) to create three-dimensional virtual curling videos as stimulus material. The videos had a resolution of 1920 × 1080 pixels, a frame rate of 30 frames per second, a focal length of 50 mm, and an initial viewing angle of 39.598 degrees. These specifications simulated the horizontal field of view of a human eye at a height of 170 cm. The footage depicted a curling stone in motion, a hog line, and the house. To avoid the possibility of a learning effect among participants due to the repetition of test materials, the curling stone was initiated from different points at varying speeds and with varying trajectories. In total, 30 distinct stimuli were prepared.

### 2.5. Speed Control Test

Prior to the speed control test, participants engaged in a standardised warm-up comprising 10 min of power cycling and a dynamic stretching routine. Subsequently, the participants proceeded to the ice rink to undertake the speed control test. During the test, the Rock Hawk timing system was set up at the delivery end of the rink with the objective of recording the time it takes for the curling stone to travel from the back line to the hog line, measured in seconds. This duration was employed to represent the range of speed control necessary for executing a slow delivery technique. The test personnel randomly selected four target values from a pre-established range (3.70–4.20 s, with 0.05-s intervals) to be used as benchmarks in the stone delivery tests. The participants commenced their delivery from the hack, with the Rock Hawk system recording the time taken for the front of each stone to travel from the back line to the hog line. The data were then immediately relayed to both the participant and the test personnel via an iPad screen. For each target value, participants delivered the curling stone on three occasions. The test personnel recorded the actual time taken for each delivery alongside the target value, with a precision of 0.01 s. The discrepancy between the exact times and the target values was used to evaluate the efficacy of the speed control mechanism.

### 2.6. Accuracy Test

Subsequently, an accuracy test was conducted following the speed control test. The delivery of the stone by the athlete is analogous to the delivery of a stone in a curling match, whereby the stone is released before reaching the hog line. A higher score is awarded the closer the stone comes to the centre of the house. Prior to the official commencement of the test, each participant was permitted two trial throws. Each athlete was required to deliver the stone in a clockwise direction on three occasions. During the official test, participants were required to utilise the same stone for each delivery. Furthermore, sweeping was not permitted as a means of adjusting the stone after its release. It was the responsibility of at least one member of the test personnel to ensure that the ice surface was cleaned before each delivery. This procedure was implemented to mitigate the potential impact of debris, such as ice chips, on the experimental results.

The scoring system for the stone’s final resting position was as follows: a score of five points was awarded if the stone successfully covered the button, four points if it landed within the one-foot ring, three points within the four-foot ring, two points within the eight-foot ring, and one point within the twelve-foot ring. In the event that the stone rested on the boundary between two rings, the score corresponding to the inner ring was awarded. From an overall perspective, the stone’s edge must enter the outer edge of a designated ring.

### 2.7. Statistical Analysis

Data were summarised and analysed using Excel 2021 and SPSS 27.0 (USA). All results are presented as mean ± standard deviation (M ± SD). Paired sample t-tests were used to compare the differences in test results under different visual conditions. A two-factor analysis of variance (ANOVA) was used to evaluate the effects of stroboscopic visual conditions and test sequence on performance in the time perception and judgment tests, as well as the curling stone delivery speed control and accuracy tests. If the main effect was significant, Bonferroni post-hoc tests were used to compare the differences between different sequences and conditions. The magnitude of the differences (effect sizes) was reported using Cohen’s *d* for paired sample t-tests. The effect size of the differences was reported using partial eta squared (η_p_^2^) and Cohen’s *f* for two-way ANOVA. A Cohen’s *d* greater than 0.8 was considered large, between 0.8 and 0.5 was categorised as medium, between 0.5 and 0.2 was considered small, and less than 0.2 was deemed insignificant. A η_p_^2^ greater than 0.14 was considered large, between 0.06 and 0.14 was categorised as medium, between 0.06 and 0.01 was considered small, and less than 0.01 was deemed insignificant. A Cohen’s *f* greater than 0.40 was considered large, between 0.40 and 0.25 was categorised as medium, between 0.25 and 0.10 was considered small, and less than 0.10 was deemed insignificant. The significance level was set at *p* < 0.05.

## 3. Results

**Figure 3:**
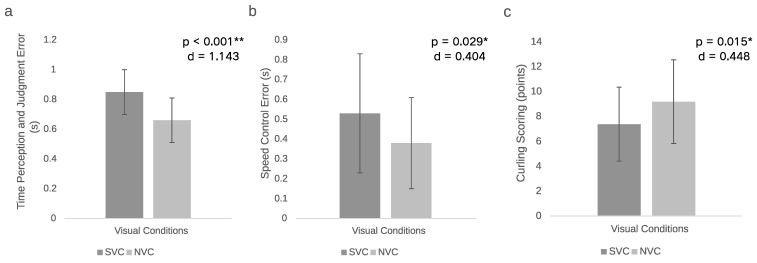
Time perception and stone delivery efficiency under different visual conditions. (**a**) Time perception and judgment error under different visual conditions (s); (**b**) speed control error under different visual conditions (s); (**c**) curling scoring under different visual conditions (points); * *p* < 0.05, ** *p* < 0.01 for differences between conditions. The data are presented as mean ± standard error (SE) in the figure.

Significant differences were observed in time perception and judgment under different visual conditions, with greater errors occurring under stroboscopic conditions (*p* < 0.001, Cohen’s *d* = 1.143, large effect size). Regarding performance in curling, the participants showed higher velocity control errors when wearing stroboscopic glasses (*p* = 0.016, Cohen’s *d* = 0.448, small effect size). In terms of accuracy, the participants under stroboscopic visual conditions scored significantly lower than under normal visual conditions (*p* = 0.029, Cohen’s *d* = 0.404, small effect size). The above results are illustrated in Table 2. The test results under different visual conditions are shown in Figure 3.

The results of the two-factor ANOVA showed that a significant main effect of the sequence was observed only in delivery speed control (*p* = 0.002, η_p_^2^ = 0.150, Cohen’s *f* = 0.420, large effect size), with no interaction effect (*p* = 0.996, η_p_^2^ = 0.000, Cohen’s *f* = 0.000, insignificant effect size). Subsequent post-hoc multiple comparisons revealed that participants who prioritised the stroboscopic glasses in testing had significantly higher speed control errors compared to the other group (*p* = 0.002, Cohen’s *d* = 0.794, medium effect size). The results of the two-factor ANOVA are shown in Table 3. The post-hoc multiple comparisons are shown in Table 4 and Table 5.

## 4. Discussion

### 4.1. Main Findings

The findings revealed that, in comparison to normal visual conditions, the stroboscopic visual conditions resulted in a notable increase in the average error of time perception and judgement among the elite curlers, a discernible rise in the error of stone delivery speed control, and a significant reduction in delivery accuracy. Furthermore, the sequence of testing had an impact on the speed control of elite athletes, although no interaction was observed between the sequence and visual conditions.

### 4.2. The Impact of Stroboscopic Visual Conditions on Cognition

It has been demonstrated that stroboscopic visual conditions negatively impact the cognitive performance of athletes. In the sport of curling, the ability to “read the ice” is of paramount importance for the successful execution of stone delivery and sweeping techniques [25]. The term refers to the process of understanding the ice conditions by observing the movement of the curling stones on the ice surface, including their slipperiness and curvature. This is considered a crucial cognitive skill in the sport [26]. The available evidence suggests that professional curling athletes possess a distinct advantage in time perception abilities in specific scenarios, characterised by the accuracy of their predictions and judgements about stone movement. This cognitive skill is closely linked to athletes’ performance in delivering stones and can, to some extent, predict their overall performance in the sport [5,27]. The presence of on-ice markings, such as the hog line, tee line, and back line, constitutes a significant environmental factor that may exert an influence on athletes’ predictions regarding stone movement during a curling match. It is important to note that the cognitive advantages observed in athletes are only evident in cognitive tests that are specifically designed to assess these skills. Consequently, the video stimuli prepared for this study accurately replicated the essential markings of a curling sheet. The experiment demonstrated that under stroboscopic visual interference, athletes exhibited significantly reduced time perception and judgement abilities in specific scenarios compared to those observed under normal visual conditions. This finding emphasises the pronounced impact of stroboscopic visual interference on these cognitive processes. The use of stroboscopic glasses, which impede the input of visual information, presented a more significant challenge for subjects engaged in time perception and judgement tasks. In accordance with prior research, the use of stroboscopic glasses has been observed to impede the processing speed of the central nervous system regarding visuomotor perception while simultaneously reducing visuomotor response speed. This may provide a neurophysiological basis for the observed performance improvements following stroboscopic training [28].

### 4.3. The Impact of Stroboscopic Visual Conditions on Stone Delivery Performance

Furthermore, stroboscopic visual conditions have an impact on athletes’ stone delivery performance, which represents the most critical specialised skill in curling. The initial velocity necessary for stone delivery is attained by exerting a force on the ice surface with the hack. The force and rhythm of the push exerted on the stone directly influence the stone’s speed and acceleration during its slide. Curlers regulate the launch speed within a specified range to guarantee that the stone acquires optimal initial velocity before it departs from their control. This enables the stone to reach a predetermined position at the far end of the track, thereby fulfilling the tactical intent as set out in reference [29]. In the majority of cases, team members use a stopwatch to accurately record and assess the speed of the stone. The most common reference point employed by athletes during competition is the time taken for the stone to traverse a fixed distance from the back line to the hog line. Sweepers may utilise this timing to anticipate the need for sweeping and the requisite intensity of said sweeping, whereas the delivering athlete may adjust the stone’s speed based on the discrepancy between the target time and the actual time. Fixed distance timing is the most commonly utilised aid in both curling training and competition [30].

The stone delivery speed control test employed in this study was designed to replicate the conditions encountered in competitive and training scenarios, wherein athletes are required to exercise precise control over the sliding speed towards a range of targets. This was achieved through the utilisation of the Rock Hawk timing system, which enabled precise recording over fixed distances. The athlete’s ability to control the speed of their delivery is dependent on their capacity to perceive time and speed in specific scenarios, which is influenced by the integration of visual, auditory, and proprioceptive input and feedback [5]. The significant reduction in visual information input under stroboscopic visual interference could also impact the athletes’ dynamic balance [31] and time perception abilities, consequently affecting both their control of delivery speed and the accuracy of their deliveries. This aligns with findings from another study involving a specialised football test, where visual feedback obstruction provided by stroboscopic glasses significantly affected performance, particularly accuracy, and had a greater impact on higher-level athletes [32]. In a separate investigation into volleyball performance, the same research team observed comparable outcomes. In this study, athletes demonstrated impaired jumping performance when exposed to stroboscopic conditions, in comparison to their performance under full-field vision. This finding suggests that incorporating stroboscopic conditions into plyometric training may be a potential avenue for further exploration [33].

### 4.4. The Potential of Stroboscopic Training in Enhancing Curling Performance

Research indicates that stroboscopic training, by limiting the input of visual information, forces athletes to perform tasks under conditions of incomplete visual feedback [28]. This scenario may prompt the brain to enhance the processing capabilities of other sensory information, such as auditory and proprioceptive inputs, thereby compensating for the deficiency in visual information [34]. Notably, under dynamic sporting conditions, the central nervous system integrates sensory information to facilitate motor control [35]. Our study’s findings suggest that delivering a curling stone under visual constraints detrimentally affects athletic performance. Evidence indicates that when either visual or proprioceptive input is restricted, the central nervous system recalibrates the contribution of individual sensory inputs [36]. This recalibration is likely more pronounced during stroboscopic training, as opposed to the reduced reliance on sensory inputs observed during steady-state movements [37]. The additional visual load introduced during training induces adaptive modifications in sensory integration, which subsequently enhances motor performance through the refinement or optimisation of sensory integration processes [38]. An increase in stroboscopic visual input may result in a reallocation of cognitive resources during training, thereby enhancing concentration and the speed of information processing. This may lead to faster reaction times and more accurate execution of movements in normal competitive environments [39]. It has been demonstrated that the curling delivery process comprises three distinct phases: the collection of information regarding the duration of the stone’s movement, the transformation and processing of motion information, and output of the delivery action [40]. The findings of this study indicate that all three stages are affected by stroboscopic visuals, suggesting that stroboscopic visuals present certain challenges to the input, processing, and output of information. As a result of restricted vision, athletes may be compelled to rely more on other senses and their intrinsic movement perception, which could lead to an overall enhancement of motor skills and cognitive abilities.

### 4.5. Prospects of this Study

This study paves the way for the potential use of stroboscopic training for elite curling athletes, with subsequent research set to further examine the impact of stroboscopic visual training on high-level curling performance. Further research could examine the effect of varying stroboscopic frequencies, athletes at different skill levels, and gender differences and compare a range of emerging training methods. The efficacy of stroboscopic training in enhancing curling performance can be gauged using a number of indicators employed in this study.

Theoretically, this study emphasises the necessity of comprehending multisensory integration in sports training and its influence on performance, demonstrating how visual information restriction impacts perception–action coupling. These findings have significant implications for the development of theories about the complex interactions between perception and action within the field of sports science. In practical terms, this study serves to bridge the gap between subsequent research and useful application. This study not only advances scientific understanding of the effects of stroboscopic conditions, but also provides a baseline for changes in curling performance under stroboscopic conditions. This will facilitate the design of subsequent studies to verify and optimise the efficacy of stroboscopic training applications.

### 4.6. Limitations of this Study

It is important to acknowledge the limitations of the present study. The relatively small sample size was primarily due to the limited availability of elite athletes and challenges in participant recruitment. Additionally, the use of a single stroboscopic frequency setting may have restricted the study’s ability to fully replicate the competitive environment, including factors such as noise and the presence of opponents. Furthermore, the results of this study may not be directly applicable to the general population of curling athletes, as only the performance of elite male curlers was measured. This study did not include biochemical or bioelectrical testing of the athletes, which future research could explore to assess the impact of stroboscopic visual conditions. Additionally, this study did not involve long-term stroboscopic training interventions, and future research could investigate the effects of such long-term training on athletic performance.

## 5. Conclusions

Stroboscopic visual interference has a significant impact on the time perception and judgement abilities of curling athletes, as well as their stone delivery performance. In conditions of stroboscopic visual interference, athletes are prone to an increase in errors in time perception and judgement, as well as a significant decline in both the control of delivery speed and accuracy. This suggests that limiting visual feedback by wearing stroboscopic glasses increases the difficulties encountered by athletes during stone delivery, thereby demonstrating the potential of stroboscopic training to improve elite curling performance.

## Figures and Tables

**Table 1:** Basic information of participants.

Sample Size	Age (Years)	Body Height (cm)	Body Mass (kg)	Training Age (Years)
*n* = 32	19.9 ± 2.2	178.0 ± 6.2	71.9 ± 10.6	2.7 ± 0.9

The data are presented as mean ± standard deviation (SD).

**Table 2:** Comparison of test results under different visual conditions.

Variable	SVC	NVC	Mean Difference	*p*	CI	Cohen’s *d*
Time Perception and Judgment Error (s)	0.85 ± 0.15	0.66 ± 0.15	0.19	<0.001 **	0.132~0.254	1.143
Speed Control Error (s)	0.53 ± 0.30	0.38 ± 0.23	0.15	0.016 *	0.029~0.264	0.448
Curling Scoring (points)	7.38 ± 2.97	9.19 ± 3.35	−1.81	0.029 *	−3.431~−0.194	0.404

The data are presented as mean ± SD; SVC: stroboscopic visual conditions; NVC: normal visual conditions; Cohen’s *d* greater than 0.8 was considered large, between 0.8 and 0.5 was categorised as medium, between 0.5 and 0.2 was considered small, and less than 0.2 was deemed insignificant; CI: 95% CI; * *p* < 0.05, ** *p* < 0.01 for differences between conditions.

**Table 3:** The impact of test sequence and visual conditions on test results.

Variable	Source	SS	df	MS	*F*	*p*	η_p_^2^	*f*
Time Perception and Judgment Error (s)	Test Sequence	0.024	1	0.024	1.076	0.304	0.018	0.135
	Visual Conditions	0.599	1	0.599	26.554	<0.001 **	0.307	0.666
	Sequence × Visual Conditions	0.038	1	0.038	1.700	0.197	0.028	0.170
Speed Control Error (s)	Test Sequence	0.687	1	0.687	10.626	0.002 **	0.150	0.420
	Visual Conditions	0.344	1	0.344	5.317	0.025 *	0.081	0.297
	Sequence × Visual Conditions	0.000	1	0.000	0.000	0.996	0.000	0.000
Curling Scoring (points)	Test Sequence	0.028	1	0.028	0.003	0.958	0.000	0.000
	Visual Conditions	61.164	1	61.164	5.999	0.017 *	0.091	0.316
	Sequence × Visual Conditions	8.601	1	8.601	0.844	0.362	0.014	0.119

η_p_^2^: partial eta squared; η_p_^2^ greater than 0.14 was considered large, between 0.06 and 0.14 was categorised as medium, between 0.06 and 0.01 was considered small, and less than 0.01 was deemed insignificant; *f*: Cohen’s *f* greater than 0.40 was considered large, between 0.40 and 0.25 was categorised as medium, between 0.25 and 0.10 was considered small, and less than 0.10 was deemed insignificant; * *p* < 0.05, ** *p* < 0.01 for differences between conditions.

**Table 4:** Post-hoc multiple comparisons for the main effect of sequence.

Variable	Sequence A	Sequence B	Mean Difference	*F*	*p*	Cohen’s *d*
Speed Control Error (s)	0.56 ± 0.30	0.35 ± 0.21	0.207	1.087	0.002 **	0.794

The data are presented as mean ± SD; Sequence A: SVC first, then NVC; Sequence B: NVC first, then SVC; Cohen’s *d* greater than 0.8 was considered large, between 0.8 and 0.5 was categorised as medium, between 0.5 and 0.2 was considered small, and less than 0.2 was deemed insignificant; ** *p* < 0.01 for differences between conditions.

**Table 5:** Post-hoc multiple comparisons for main effect between groups.

Variable	SVC	NVC	Mean Difference	*F*	*p*	Cohen’s *d*
Time Perception and Judgment Error (s)	0.85 ± 0.15	0.66 ± 0.15	0.193	26.226	<0.001 **	1.280
Speed Control Error (s)	0.53 ± 0.30	0.38 ± 0.23	0.147	4.668	0.035 *	0.540
Curling Scoring (points)	7.38 ± 2.97	9.19 ± 3.35	−1.813	5.253	0.025 *	−0.573

The data are presented as mean ± SD; SVC: stroboscopic visual conditions; NVC: normal visual conditions; Cohen’s *d* greater than 0.8 was considered large, between 0.8 and 0.5 was categorised as medium, between 0.5 and 0.2 was considered small, and less than 0.2 was deemed insignificant; * *p* < 0.05, ** *p* < 0.01 for differences between conditions.

## Data Availability

The original contributions presented in this study are included in the article/Supplementary Material, further inquiries can be directed to the corresponding author/s.

## Supplementary Materials

The following supporting information can be downloaded at: https://www.mdpi.com/article/10.3390/life14091184/s1.
