# Supplementary material for: The Impact of Stroboscopic Visual Conditions on the Performance of Elite Curling Athletes
Source: Life (Basel). 2024 Sep 19;14(9):1184. doi: 10.3390/life14091184 (PMC11432937; doi:10.3390/life14091184)
Supplement: Supplementary file 1 [file life-14-01184-s001.zip › Informed Consent Form (CHN and ENG).pdf]

Informed Consent Form (ENG)

Dear Participant,

Greetings! First of all, thank you for considering participating in the research project "The Impact of Stroboscopic Visual Conditions on the Performance of Elite Curling Athletes" conducted by Beijing Sport University.

Purpose and Content of the Research:

This study aims to explore the impact of different training methods on the accuracy of stone-throwing, time, and speed perception abilities of curling athletes. The study will last for four weeks, during which you will be randomly assigned to three groups for four weeks of training under specific conditions, and two tests will be conducted before and after the training (including pitching performance, time, and speed perception).

Inclusion and Exclusion Criteria:

Participants must be athletes of national first-level ranking or above.

Participants must be in good health, with no major illnesses.

Participation is voluntary, and you must sign this Informed Consent Form before the commencement of the experiment.

Participant Rights:

Your participation is entirely voluntary, and you may withdraw at any time without any negative consequences.

Your personal information will be strictly confidential and used only for the purposes of this research.

Upon completion of the study, you are entitled to know the overall results of the research.

Risks and Discomforts:

The risk associated with this study is very low. You may experience fatigue or slight discomfort during training. Should you feel any discomfort, please stop training immediately and notify the research personnel.

Basic Information Survey:

Before participating in this study, you will be required to fill out a "Basic Information Questionnaire," which includes, but is not limited to, your name, age, place of birth, medical history, family history, years of training, athletic level, and sports experience.

Contact Information:

If you have any questions or need assistance, you can contact the project leader, Tianhe Li, at any time. *Phone: 13701033517*, *Email: 2252588618@qq.com*.

Consent Statement:

I *have fully understood the purpose, procedures, and potential risks of this research and agree to participate voluntarily. I understand that I can withdraw from the study at any time, and my withdrawal will not have any negative impact on me.*

Participant Signature: ________________ Date: __________
